# An efficient genome sequencing method for equine influenza [H3N8] virus reveals a new polymorphism in the PA-X protein

**DOI:** 10.1186/1743-422X-11-159

**Published:** 2014-09-02

**Authors:** Adam Rash, Alana Woodward, Neil Bryant, John McCauley, Debra Elton

**Affiliations:** Animal Health Trust, Lanwades Park, Kentford, Newmarket CB8 7UU UK; MRC National Institute for Medical Research, Mill Hill, London UK

**Keywords:** Equine influenza virus, H3N8, Genome sequencing, Non-coding regions, M13, PA-X

## Abstract

**Background:**

H3N8 equine influenza virus (EIV) has caused disease outbreaks in horses across the world since its first isolation in 1963. However, unlike human, swine and avian influenza, there is relatively little sequence data available for this virus. The majority of published sequences are for the segment encoding haemagglutinin (HA), one of the two surface glycoproteins, making it difficult to study the evolution of the other gene segments and determine the level of reassortment occurring between sub-lineages.

**Methods:**

To facilitate the generation of full genome sequences for EIV, we developed a simple, cost-effective and efficient method. M13-tagged primers were used to amplify short, overlapping RT-PCR products, which were then sequenced using Sanger dideoxynucleotide sequencing technology. We also modified a previously published method, developed for human H3N2 and avian H5N1 influenza viruses, which was based on the ligation of viral RNA and subsequent amplification by RT-PCR, to sequence the non-coding termini (NCRs). This necessitated the design of novel primers for an N8 neuraminidase segment.

**Results:**

Two field isolates were sequenced successfully, A/equine/Lincolnshire/1/07 and A/equine/Richmond/1/07, representative of the Florida sublineage clades 1 and 2 respectively. A total of 26 PCR products varying in length from 400–600 nucleotides allowed full coverage of the coding sequences of the eight segments, with sufficient overlap to allow sequence assembly with no primer-derived sequences. Sequences were also determined for the non-coding regions and revealed cytosine at nucleotide 4 in the polymerase segments. Analysis of EIV genomes sequenced using these methods revealed a novel polymorphism in the PA-X protein in some isolates.

**Conclusions:**

These methods can be used to determine the genome sequences of EIV, including the NCRs, from both clade 1 and clade 2 of the Florida sublineage. Full genomes were covered efficiently using fewer PCR products than previously reported methods for influenza A viruses, the techniques used are affordable and the equipment required is available in most research laboratories. The adoption of these methods will hopefully allow for an increase in the number of full genomes available for EIV, leading to improved surveillance and a better understanding of EIV evolution.

**Electronic supplementary material:**

The online version of this article (doi:10.1186/1743-422X-11-159) contains supplementary material, which is available to authorized users.

## Background

Equine influenza virus (EIV) is an influenza A virus belonging to the *Orthomyxoviridae* family. These viruses have a negative sense, single-stranded RNA genome consisting of eight viral gene segments [1]. Originally thought to have transmitted from birds, H3N8 EIV was first isolated during a widespread outbreak in the United States in 1963 [2], and has since spread worldwide causing multiple major outbreaks of disease in horses. During the 1980s the virus diverged into two antigenically distinct lineages [3], American and Eurasian, and since then the American lineage has further evolved into the Florida sublineage clades 1 and 2, which continue to co-circulate today [4]. These lineages have historically been based on antigenic and genetic data for HA. A phylogenetic study by Murcia et al. [5] showed that phylogenetic trees produced for each of the viral gene segments also supported division into the American and Eurasian lineages, and all but segment 7 divided into the two clades of the Florida sublineage. However, less than 100 complete viral genomes covering the 46 years from 1963 to 2008 were available at the time of the study.

Other groups have studied the evolution of individual influenza A virus genes. H3N8 EIV PB2 [6] and matrix proteins [7] were found to belong to the same lineage as North American avian strains, whilst PB1, PA, HA and NP were found to have evolved independently from other influenza A viruses [8–10]. Equine NS was suggested as being restricted to subtype [11], as the NS segments of the H3N8 viruses were close to one another but not to that of the H7N7 viruses, however very limited numbers of EIV genomes were available at the time these studies were performed, and in some cases only two different EIV strains were used. More recently a study found that the internal genes of the 1963 EIV pandemic virus were of western hemispheric avian influenza origin [12]. This study also showed that the virus shared a most recent common ancestor with avian influenza viruses from South America shortly before its emergence. The time to most recent common ancestor for avian/equine NP was calculated as being 1954, which agreed with the hypothesis that the virus emerged in South America prior to its introduction into the USA in 1963 by horses imported by air from Argentina [12].

Following an extensive outbreak in 1989 affecting a highly vaccinated population of racehorses in the UK, it became clear that, like human influenza virus, EIV undergoes antigenic drift and therefore vaccine strains need to be kept up to date [13, 14]. A formal process for vaccine strain selection, overseen by the World Organisation for Animal Health (OIE) was put in place. This process relies heavily on surveillance data collected from the field, of which most is focussed solely on the HA gene and the protein it encodes. Therefore the majority of published sequences are for HA, which makes it difficult to study the evolution of either the other gene segments or the virus as a whole. It is also known that reassortment between the different lineages of EIV has occurred [4–8, 15, 16] but the full extent is unknown due to a lack of data available. Avian H3N8 influenza viruses have been shown to frequently exchange internal gene segments, and it has been suggested that the extensive reassortment within the H3 subtype poses a threat to human and animal health [17]. Next generation sequencing technologies have made it easier to sequence whole viral genomes, however these technologies are not readily available to all as considerable investment in equipment and bioinformatics expertise are needed. We aimed to develop a simple and robust method to sequence whole EIV genomes from all H3N8 lineages using Sanger dideoxynucleotide sequencing technology.

Each of the eight influenza virus gene segments contains two non-coding regions (NCRs), one at the 5′ terminus containing 13 conserved nucleotides, and the other at the 3′ terminus, which contains 12 nucleotides [18]. Unlike the 5′ end, the 3′ terminus exhibits variation at the fourth nucleotide. This variation in the fourth nucleotide has been shown to affect the rescue of virus from a reverse genetics system [19]. A second objective, to implement a method previously described for sequencing the NCRs of influenza viruses [20], was adapted and carried out on an EIV, as well as an N8 subtype neuraminidase, for the first time.

Here we describe the genome sequencing method and highlight the sequence differences found between representatives of the two circulating clades of the Florida sublineage.

## Results

### Genome sequencing of equine influenza viruses

**Figure 1:**
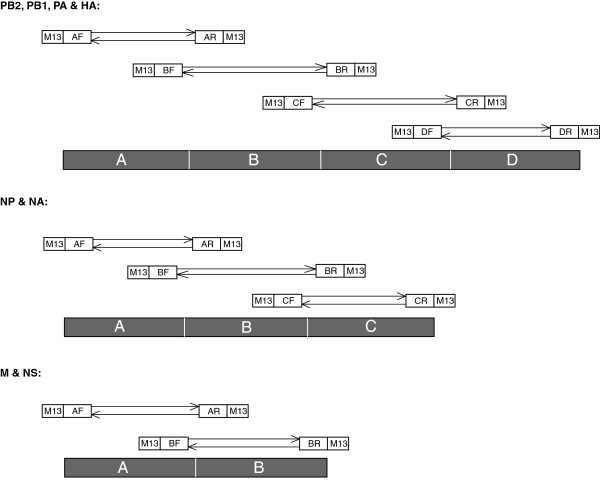
**Schematic representation of PCR primer design for genome sequencing EIV.** Primers were tagged with M13 forward or reverse sequences for use in the sequencing stage. Segments 1–4 (PB2, PB1, PA and HA) were divided into four sections, segments 5 & 6 (NP and NA) into three sections and segments 7 & 8 (M and NS) into two sections, each of approximately 400–600 nucleotides in length.

**Figure 2:**
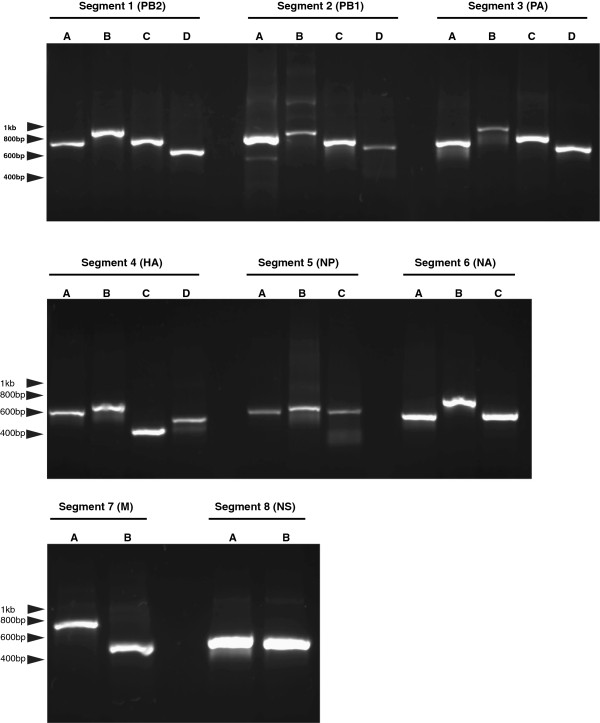
**Agarose gel electrophoresis analysis of genome segment PCR products.** Agarose gel (1%) showing PCR fragments A, B, C & D of segments 1–4 (PB2, PB1, PA and HA), A, B & C of segments 5 and 6 (NP and NA), and A & B of segments 7 and 8 (M and NS) of EIV Northamptonshire/1/13.

At the time of writing, only 81 full genome sets were available from the NCBI Influenza Virus Resource for EIV and only one or two gene segment sequences had been published for the majority of strains. To address the lack of available genomes, a method to sequence the genomes of equine influenza viruses belonging to both clades of the currently circulating Florida sublineage, using an EIV specific primer set for PCR and M13 primers for sequencing, was developed. A/equine/Richmond/1/07 was selected as a representative of recent Florida sublineage clade 2 (FC2) viruses, as well as being a current OIE recommended vaccine strain. A/equine/Lincolnshire/1/07 was chosen because it was the first virus belonging to clade 1 of the Florida sublineage isolated in the UK [4]. Published nucleotide sequences were aligned for each segment and primers were designed to conserved regions (data not shown) to amplify products of 400–600 nucleotides (Figure 1). Each specific primer was elongated at the 5′ end by adding either M13 forward or M13 reverse primer sequences (Table 1), as described in a method for sequencing swine influenza genomes [21] which facilitated efficient sequencing. Four amplicons were produced for the larger genome segments, 1 to 4, three amplicons for segments 5 and 6, and two amplicons for segments 7 and 8 (Figure 2). A total of 26 PCR products were amplified successfully from RNA extracted from allantoic fluid for both virus strains, A/equine/Richmond/1/07 and A/equine/Lincolnshire/1/07. The nucleotide sequences of the PCR products were determined on both strands and the complete viral genome was assembled successfully without the need for further specific primers to complete gaps. The PCR products overlapped by approximately 100 nucleotides at each end and sequences were edited, so that the final coding sequence contained no primer-derived sequences. This method has since been applied to a further 17 strains of EIV, all primers worked well for Florida clade 1 and clade 2 strains from 2009–2013, and sequences were made available on the GISAID (Global Initiative on Sharing Avian Influenza Data) EpiFlu database [22] (see Additional file 1). Assembled sequences for each segment of A/equine/Richmond/1/07 and A/equine/Lincolnshire/1/07 were also uploaded onto GenBank, accession numbers indicated in additional material (see Additional file 2).

**Table 1:** **Primer sequences and annealing temperatures used to sequence the genome of H3N8 EIV**

Primer name	Primer sequence (5′-3′)	Approximate nucleotide coverage (5′-3′)	Annealing temperature used (°C)
PB2/AF	GC GTAAAACGACGGCCAGT AGCGAAAGCAGGCAAATATATTCAATATG	1-655	50
PB2/AR	GC AACAGCTATGACCATG CTCTTTCTAGCATGTAT		
PB2/BF	GC GTAAAACGACGGCCAGT CACAACTAACAATAACCAA	569-1335	60
PB2/BR	GC AACAGCTATGACCATG CCTCAAGAGTTGATG		
PB2/CF	GC GTAAAACGACGGCCAGT GCAATAATTGTAGCC	1216-1874	45
PB2/CR	GC AACAGCTATGACCATG ATTATTTGAGCAGTATC		
PB2/DF	GC GTAAAACGACGGCCAGT GAAGCCAATACAGCGGT	1793-2341	50
PB2/DR	GC AACAGCTATGACCATG AGTAGAAACAAGG TCGTTTTTAAACAATTC		
PB1/AF	GC GTAAAACGACGGCCAGT AGCGAAAGCAGG CAAACCATTTGAATGG	1-719	50
PB1/AR	GC AACAGCTATGACCATG CAGCGTCCTTGGTCATTG		
PB1/BF	GC GTAAAACGACGGCCAGT CTTCCAACGAAAGAGAA	577-1301	50
PB1/BR	GC AACAGCTATGACCATG GGTTTAATATGGATACACC		
PB1/CF	GC GTAAAACGACGGCCAGT GCGGCTTCACTGAGTCCTGGC	1222-1863	50
PB1/CR	GC AACAGCTATGACCATG CATTTTAAACAAACTTC		
PB1/DF	GC GTAAAACGACGGCCAGT CAAAGACTGGTCTACTG	1789-2341	50
PB1/DR	GC AACAGCTATGACCATG AGTAGAAACAAGG CATTTTTTCATGAAGATC		
PA/AF	GC GTAAAACGACGGCCAGT AGCAAAAGCAGG TACTGATCCAAAATGG	1-615	50
PA/AR	GC AACAGCTATGACCATG GCCTCTCTCGGACTGAC		
PA/BF	GC GTAAAACGACGGCCAGT GCCAGAATCAAGACCAGG	529-1255	50
PA/BR	GC AACAGCTATGACCATG CTCACTTGGAATCCAACTTGC		
PA/CF	GC GTAAAACGACGGCCAGT GAGAGAAAGTGGATTTTGAGGATTG	1149-1785	50
PA/CR	GC AACAGCTATGACCATG CTGAAGGAGGCAGCGCC		
PA/DF	GC GTAAAACGACGGCCAGT GACCCATGTTTTTGTATG	1700-2233	50
PA/DR	GC AACAGCTATGACCATG AGTAGAAACAAGG TACTTTTTTGGACAG		
HA/AF	GC GTAAAACGACGGCCAGT AGCGAAAGCAGGGGACGATATT	1-515	50
HA/AR	GC AACAGCTATGACCATG GATTTGTTAGCCAATTCAG		
HA/BF	GC GAAAACGACGGCCAGT CAGGTGTCACTCAAAAC G	428-1032	50
HA/BR	GC AACAGCTATGACCATG GGATTTGCTTTTCTGGTAC		
HA/CF	GC GTAAAACGACGGCCAGT GGTTACATATGGAAAATGCC	939-1336	50
HA/CR	GC AACAGCTATGACCATG GAGCCACCAGCAATTCT		
HA/DF	GC GTAAAACGACGGCCAGT GAAGGAAGAATTCAGGA	1251-1733	50
HA/DR	GC AACAGCTATGACCATG GAGTAGAAACAAGGGTGTTTTTAAC		
NP/AF	GC GTAAAACGACGGCCAGT AGCGAAAGCAGGGTAGATAATC	1-570	50
NP/AR	GC AACAGCTATGACCATG CCGTGGGAGGGTTGAGCC		
NP/BF	GC GTAAAACGACGGCCAGT GACACCACATACCAAAC	480-1075	45
NP/BR	GC AACAGCTATGACCATG CTCTCAGGTCCTCAAAT		
NP/CF	GC GTAAAACGACGGCCAGT CCAGCACACAAGAGCCAG	1012-1569	55
NP/CR	GC AACAGCTATGACCATG AGTAGAAACAAGGGTATTTTTC		
NA/AF	GC GTAAAACGACGGCCAGT AGCAAAAGCAGGAGTTT	1-508	45
NA/AR	GC AACAGCTATGACCATG GCCCTATTTTGACACTC		
NA/BF	GC GTAAAACGACGGCCAGT CACACAGGGCTCATTAC	417-1049	45
NA/BR	GC AACAGCTATGACCATG CCGAAACCTTTTACACCG		
NA/CF	GC GTAAAACGACGGCCAGT CACAGTTGGATATTTGTG	951-1461	50
NA/CR	GC AACAGCTATGACCATG AGTAGAAACAAGGAGTT		
M/AF	GC GTAAAACGACGGCCAGT AGCGAAAGCAGGTAGATATTTAAAG	1-654	50
M/AR	GC AACAGCTATGACCATG CTAGCCTTACTAGCAAC		
M/BF	GC GTAAAACGACGGCCAGT CAGTACCACGGCTAAAG	571-1027	50
M/BR	GC AACAGCTATGACCATG AGTAGAAACAAGGTAGTTTTTTAC		
NS/AF	GC GTAAAACGACGGCCAGT AGCGAAAGCAGGGTGACAAAAAC	1-492	50
NS/AR	GC AACAGCTATGACCATG CTGCTCCTTCTTCGGTG		
NS/BF	GC GTAAAACGACGGCCAGT CATCATACTTAAAGCAAAC	407-890	50
NS/BR	GC AACAGCTATGACCATG AGTAGAAACAAGGTAGTGTTTTTTAT		

The gene segment sequences for each of the two viruses were aligned against one another for comparison. There were a total of 279 nucleotide differences between the two viruses, which resulted in 65 amino acid changes (Table 2). Approximately 45% (29) of the total amino acid changes were observed within the polymerase and nucleoprotein segments, with 45% (13) of these occurring within PA alone. The two glycoprotein segments, HA and NA, contained 40% (26) of the total amino acid differences, of which 54% (14) were within HA and 46% (12) within NA.

**Table 2:** **Nucleotide and amino acid differences between A/equine/Richmond/1/07 and A/equine/Lincolnshire/1/07**

Segment	Nucleotide changes	Protein	Amino acid changes	Amino acid changes from Richmond/1/07 to Lincolnshire/1/07
1	35	PB2	4	A105T, K251R, I398V, K660R
2	48	PB1	8	F94L, M119V, V149I, M179I, R329Q, E377D,D618E, K621R
3	47	PA	13	D64E, I86M, M210A, K237E, G240E, P259S, N321S, L348I, S409N, I465V, T476A, I500V, R626K
4	47^a^	HA1^†^	11^b^	K-14T, F-11L, I-9a^1^, F-9b^1^, N7G, R62K, V78A, D104N, A138S, N159S, E291D
		HA2	3	T43A, E50G, I198V
5	30	NP	4	I131M, T257I, A359T, S450N
6	37	NA^‡^	12	T9A, S12F, V35A, E40K, G42D, H66Y, P78S, I191V, N235D, S337N, I410V, G416E
7	24	M1	3	I15V, I80V, K95R
		M2	2	S86D, 290G
8	11	NS1	4	I48S, I84V, Y207H, G210R
		NS2/NEP	1	M52I

^a^Includes a 6 nucleotide duplication resulting in ^b^two additional amino acids in A/equine/Richmond/1/07. ^†^Numbering starting after the putative signal sequence, ^‡^numbering starting from start codon ^1^Two amino acid insertion in Richmond/1/07 HA, not present in Lincolnshire/1/07.

The two smaller segments, M and NS, contained a total of 10 amino acid changes within the four predicted polypeptides (M1, M2, NS1 and NS2/NEP) that they encode.

Nucleotide changes in three segments resulted in different lengths for their predicted polypeptides. A duplication of six nucleotides in A/equine/Richmond/1/07 resulted in a two amino acid insertion within the putative signal peptide of the precursor HA protein extending its length from 15 to 17 amino acids, as observed in recent FC2 isolates [4]. In contrast, when compared to earlier EIV isolates from 1963–2000 the predicted amino acid sequences for the NS1 protein from both viruses were truncated by 11 amino acids, as seen for other recent isolates [4]. This was caused by a premature stop codon at position 220, resulting in a predicted polypeptide length of 219 amino acids. The open reading frame of NS2/NEP however, was unaffected by this nucleotide substitution. In addition, a novel truncation in the recently discovered PA-X gene was identified in A/equine/Richmond/1/07, caused by an early stop codon at position 20 of the +1 reading frame. The truncation of PA-X by 42 amino acids has not been described before, with the majority of strains having either a full length version (252 amino acids) or are truncated by 19 amino acids. To investigate further and to study the evolution of the truncation, the PA-X region of segment 3 from an additional 29 EIV isolated in the UK between 2005 and 2013, including 9 from 2007, were sequenced using the method described here (see Additional file 3). The 42 amino acid truncation in A/equine/Richmond/1/07 PA-X was identified in three of the isolates, one from another horse in the same outbreak that A/equine/Richmond/1/07 was isolated from, and the other two from a separate outbreak in 2007. The remaining isolates did not share the truncation, and all had a full length PA-X of 252 amino acids (61 amino acids following the +1 frameshift) (see Additional file 3).

### Sequencing of the non-coding regions of equine influenza viruses

**Figure 3:**
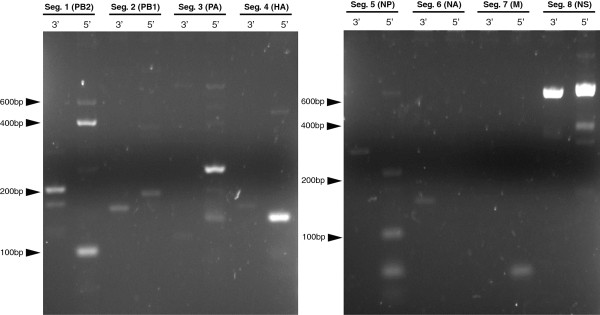
**Agarose gel electrophoresis analysis of non-coding region PCR products.** Agarose gel (2.5%) showing PCR fragments for the non-coding regions of A/equine/Richmond/1/07 influenza virus gene segments. The positions of molecular weight markers are indicated by black arrows. Bands of the expected size were visible in all lanes except for NA 5′, M 3′ and 5′.

Previous studies have shown that there are discrepancies in the segments that contain a cytosine at nucleotide position four of the 3′ NCR, and that a cytosine at this position is not restricted to the polymerase segments [23]. This variation has been implicated in the differing levels of vRNA and mRNA synthesis observed during the virus replication cycle whereby a uracil at this position increased mRNA production and delayed vRNA synthesis [23]. Another study showed that the fourth nucleotide of the NCR at the 3′ end of influenza vRNA segments could influence rescue of viruses using reverse genetics [19]. As one of our future aims was to generate a reverse genetics system for A/equine/Richmond/1/07, we determined the sequence of the NCRs for each vRNA segment for this virus strain. Viral RNA was self-ligated, then each of the NCRs amplified using a universal primer complementary to the opposite NCR and a segment specific primer, as described by de Wit et al. [20]. Modifications were made to all but two of the published primer sequences to ensure that they were complementary to equine influenza viruses sequenced previously (Table 3). The NCRs from each segment were amplified successfully, but with varying degrees of efficiency. In particular the 3′ of segment 8 was amplified to a high level, whereas products of the correct size for the 5′ of segment 6, and both the 5′ and 3′ of segment 7 were not visible by gel electrophoresis, as shown in Figure 3. Despite bands of the correct size not being visible for these products, sequence covering the NCR regions of interest were successfully determined for all three. Subsequent sequencing of the amplified NCRs revealed that the 13 nucleotides of the 5′ end were identical in all 8 viral gene segments, as well as the 12 bases of the 3′ end of the vRNA except for the fourth nucleotide. The three polymerase segments (PB2, PB1 and PA) all contained a cytosine, whilst the remaining segments (HA, NP, NA, M and NS) contained a uracil at the fourth nucleotide position.

**Table 3:** **Primer sequences used for sequencing 3′ and 5′ NCRs of H3N8 EIV**

Primer	Primer sequence ^†^
Universal 3′	5′-*CCTTGTTTCTACTAGC*-3′
Universal 5′	5′-*CCTGCTTTTGCTAGT*-3′
PB2 3′	5′-*GG* **G** *TATTTCATTGCCATCATCC*-3′
PB2 5′	5′-*GACTCTAGCATACTTACTGACAG*-3′
PB1 3′	5′-*GAC* **A** *GT* ***A*** *TCCATGGTGTATCCTGT*-3′
PB1 5′	5′-*G* **T** *ATGGT* **T** *GAGGCCATGGTGTC* **C**-3′
PA 3′	5′-**A** *T* **C** *CCTGTGATTTCAAATCTTTCTTC*-3′
PA 5′	5′-*GA* **A** *TGCCTGATTAATGATCCCTG*-3′
HA 3′	5′-*A* **AT** *GTTCC* **A** *TTTG* **CT** *ACTGCATG*-3′
HA 5′	5′-*GGATTTC* **A** *TT* **C** *GCCATATCATG*-3′
NP 3′	5′-*TCAAA* **T** *GC* **C** *GA* **A** *AG* **T**-3′
NP 5′	5′-*CCGATCGTGCC* **T** *TC* **C** *TTTGACA*T-3′
NA 3′	5′-GTGGAGTAGATCATAAAATTGCC-3′
NA 5′	5′-CAGACCTGTTTCATTGTTATTGAG-3′
M 3′	5′-*A* **T** *AAAGCGTCTACGCTGCAGTCC*-3′
M 5′	5′-*AAAGAGGGCCTTCTACGGAAGG*-3′
NS 3′	5′-*C* **C** *GT* **A** *TTATCATTCCATT* **T** *AAG*-3′
NS 5′	5′-*TGATAA* **T**AC**G**GTT**A**GA**A**TCTCT-3′

^†^Sequences from de Wit et al., [20] shown in italics with specific individual nucleotide changes in bold text, plus novel primer sequences for the N8 NA segment.

## Discussion

We and others have previously shown that reassortment has occurred between different EIV [4–8, 15, 16], but a lack of full genome sequences for EIV makes it difficult to ascertain the extent of reassortment between them, and whether reassortment has occurred between EIV and influenza A viruses from other species. We therefore developed a simple method for sequencing viral genomes that included primers with M13 sequence tags to improve the sequencing efficiency. This was based upon the approach recommended by the WHO for sequencing swine influenza virus isolates in 2009 [21], however our method used only 26 PCR fragments to cover the segment-specific regions of EIV, rather than 46 fragments. Alternative methods have been employed for sequencing influenza A viruses, such as using universal primers to simultaneously amplify all eight genome segments, or segment specific primers to amplify entire segments; however, in our hands such protocols result in poor amplification of the three largest genome segments (data not shown). Other methods based on amplification of small PCR fragments do not include the M13 sequences in the primers, which makes the method described here simple and efficient. A method previously described for sequencing the NCRs of the influenza gene segments was also modified and successfully used for the first time on an equine influenza virus, with novel primers designed for an N8 subtype NA.

The two segments encoding the surface glycoproteins, HA and NA, contained a large number of amino acid differences between the two viruses. This was expected as these two proteins are under constant immune-driven selection pressure to undergo antigenic drift. Interestingly a high number of amino acid differences were found in PA, especially when compared to the other two polymerase subunits PB2 and PB1, and a similar finding was observed by Murcia et al. [5]. The other internal segments contained fewer changes, which is not surprising as they are both smaller and may be under less immune pressure than the surface proteins.

Interestingly, a mutation in the +1 reading frame of PA, causing a premature stop codon in the translated amino acid sequence of PA-X, was observed in A/equine/Richmond/1/07. PA-X is a recently discovered protein containing the N-terminal 191 amino acids of PA and, in the majority of strains, a further 61 amino acids derived from a frameshift to the +1 reading frame of PA [24]. PA-X has been implicated in the modulation of influenza virus pathogenicity and virulence in a mouse model, whereby PA-X deficient viruses caused greater clinical signs and were less able to shut off host cell responses compared to wild-type viruses with full length PA-X [24]. The premature stop codon in A/equine/Richmond/1/07 would lead to a truncation of the protein by 42 amino acids. Truncated forms of PA-X have been described previously, however the majority of these are due to a nonsense mutation at codon 42 in the +1 reading frame [25]. Sequencing of PA, as described here, revealed that several other virus isolates from different outbreaks in 2007 as well as from the same yard as A/equine/Richmond/1/07, had the same truncated form of PA-X, however the truncated form did not persist in the UK.

Sequence analysis of the NCRs from each segment showed that EIV strain A/equine/Richmond 1/07 had cytosine at position 4 of the 3′ vRNA in the three polymerase segments, as found in other influenza viruses, and uracil at this position in the remaining 5 segments. This is the same pattern seen in the majority of other influenza A viruses for which the promoter sequences have been determined, including the prototype avian influenza virus, A/chicken/Rostock/34 (H7N1) [26].

The methods outlined here can be used to determine the genome sequences of EIV, including the NCRs, from both clade 1 and clade 2 of the Florida sublineage. The techniques described here are affordable, and the equipment required is available in most research laboratories. The sequence assembly process is simple and does not require in depth bioinformatics, unlike next generation sequencing methodology. Due to the small genome size and small sample numbers usually associated with EIV, this method is therefore highly cost effective and straightforward. Amplicon sequencing has also been shown to be less labour intensive and more affordable than plasmid cloning methods [27]. This method also permits the sequencing of individual gene segments with relative ease, as was the case with PA described here to investigate the frequency of the truncated form of PA-X.

## Conclusions

We have developed a simple, efficient and affordable method for sequencing whole genomes of EIV that offers an improvement compared with previously published methods. The adoption of these methods should facilitate an increase in the number of full genome sequences available for EIV. This will benefit surveillance programmes for EIV and improve understanding of the evolutionary paths taken by the virus, including the level of reassortment.

## Methods

### Viruses

EIV A/equine/Richmond/1/07 and A/equine/Lincolnshire/1/07 had previously been isolated and passaged twice in embryonated chicken eggs [4]. RNA was isolated from 140 μl virus stocks containing ~10^7^ EID_50_/ml using a QIAamp Viral RNA Mini Kit (Qiagen), according to manufacturer’s directions. RNA was eluted in 50 μl elution buffer.

### cDNA synthesis

Using a UNI-12 primer, as described by Hoffmann et al. [28], cDNA was transcribed by denaturing 2 μl RNA in the presence of UNI-12 (1 μM final concentration) and 7 μl water at 70°C for 10 minutes and then cooling on ice. Following this, dNTP mix (final concentration each 0.5 μM), 1 × First Strand buffer, 200U Superscript II reverse transcriptase (Invitrogen) and water to a final volume of 20 μl were added. The reaction mixture was incubated at 42°C for 45 minutes.

### PCR amplification of gene segments

Viral gene segments were amplified in 50 μl PCR reactions consisting of 2 μl cDNA (representing 10% of the reverse transcription reaction), dNTP mix (0.2 mM each final concentration) (Qiagen), 1 × Pfu buffer, 2.5U Native Pfu DNA polymerase (Stratagene), water and oligonucleotide pairs (final concentration each of 0.2 μM) as listed in Table 1. The cycling conditions were as follows: initial denaturation at 96°C for 1 minute, followed by 25 cycles of denaturation at 96°C for 15 seconds, primer annealing at 50-60°C (see Table 1) for 10 seconds and elongation at 60°C for 5 minutes. PCR reactions were analysed on a 1% agarose gel containing GelRed nucleic acid stain (Biotium) according to manufacturer’s directions. PCR products were purified using a QIAquick PCR purification kit (Qiagen) according to manufacturer’s directions.

### PCR amplification of non-coding regions

The method described by de Wit et al. [20] was used with modifications, as detailed in Table 2. Novel primers were designed to amplify the N8 subtype NA segment. Briefly, following an initial denaturation at 65°C for 5 minutes in the presence of T4 RNA ligase buffer and 20U RNAsin RNase inhibitor (Promega), 15 μl RNA was ligated using 40U T4 RNA ligase (New England Biolabs) at 37°C for 1 hour. The ligation reaction was stopped by heat inactivation at 65°C for 10 minutes. cDNA was transcribed from 4 μl of the ligated RNA by incubating the RNA in a mixture consisting of 0.5 μg random primer (Promega), dNTP mix (0.5 mM each final concentration) (Qiagen) and 20U RNasin at 65°C for 5 minutes, then cooling to 4°C, before adding 20U RNasin, 5 mM DTT, 200U Superscript II (Invitrogen) and 1 × First Strand buffer in a total reaction volume of 20 μl. The reaction mixture was subsequently incubated at 25°C for 5 minutes, followed by 50°C for 1 hour. 50 μl PCR reactions consisting of 4 μl cDNA (representing 20% of the reverse transcription reaction), dNTP mix (20 mM final concentration), universal primer (3′- or 5′- final concentration 0.2 μM) (Table 2), gene segment specific primer (3′- or 5′- final concentration 0.2 μM) (Table 2), 1 × Pfu buffer and 2.5U Native Pfu DNA polymerase (Stratagene) were made. The cycling conditions were as follows: initial denaturation at 96°C for 6 minutes, followed by 40 cycles of denaturation at 96°C for 30 seconds, primer annealing at 37°C for 1 minute and elongation at 72°C for 2 minutes. PCR reactions were analysed on a 2.5% agarose gel containing GelRed nucleic acid stain (Biotium) according to manufacturer’s directions. PCR products were purified using a QIAquick PCR purification kit (Qiagen) according to manufacturer’s directions. Where multiple bands were present in the gel, bands of the correct size were excised and purified using a QIAquick gel extraction kit (Qiagen) according to manufacturer’s directions.

### Sequencing

Sequencing reactions were performed using the BigDye terminator sequencing kit version 3.1 (Applied Biosystems). M13 forward and reverse primers were used for gene segment PCR products, whilst for the non-coding regions the primers used for the PCR stage were reused, both at a final concentration of 80nM. The sequencing reactions were run on a 3130xl genetic analyzer (Applied Biosystems), and the resulting nucleotide sequences were visualised, assembled and edited using SeqMan II version 5.03 (DNAStar, Inc) and BioEdit version 7.0.5.3 (Ibis Pharmaceuticals Inc.).

## Electronic supplementary material

Additional file 1: **GISAID EpiFlu database [** [19]**] accession numbers for PA-X sequences.** (DOCX 21 KB)

Additional file 2: **GenBank accession numbers for A/equine/Richmond/1/07 and A/equine/Lincolnshire/1/07, and GISAID EpiFlu database [** [19]**] accession numbers for other strains.** (DOCX 14 KB)

Additional file 3: **Alignment of predicted amino acid sequences for PA-X from EIV isolated in the UK between 2005 and 2013.** Amino acids of the C-terminal PA-X domain only, following the +1 frameshift, are shown. (DOCX 15 KB)
