# Supplementary material for: An efficient genome sequencing method for equine influenza [H3N8] virus reveals a new polymorphism in the PA-X protein
Source: Virol J. 2014 Sep 2;11:159. doi: 10.1186/1743-422X-11-159 (PMC4161859; doi:10.1186/1743-422X-11-159)
Supplement: Supplementary file 1 — Additional file 1: GISAID EpiFlu database [ [19]] accession numbers for PA-X sequences. (DOCX 21 KB) [file 12985_2014_2483_MOESM1_ESM.docx]

Additional file 1

.

| Isolate | Accession number |
| --- | --- |
| Lanark/05 | EPI507486 |
| Wales/05  Essex/1/05  Essex/2/05  Lanark/06  Southampton/06  Lincolnshire/06  Horsham/07 | EPI507483  EPI507484  EPI507485  EPI507488  EPI507487  EPI507489  EPI507490 |
| Maidstone/1/2007 | EPI504537 |
| Southampton/1/2007 | EPI504538 |
| Southampton/2/2007 | EPI504539 |
| Richmond/2/2007 | EPI503957 |
| Newmarket/2007 | EPI504540 |
| Cheshire/1/2007 | EPI504541 |
| Cheshire/2/2007 | EPI504542 |
| Cheshire/3/2007  Aboyne/2/08  Lanarkshire/1/08  Lanarkshire/2/08  Lanarkshire/3/08  Perthshire/3/09  Yorkshire/3/09  Dorset/09  Lanarkshire/09  Shropshire/10  Devon/1/11  East Renfrewshire/2/11  Northamptonshire/1/13 | EPI504543  EPI507491  EPI507492  EPI507493  EPI507494  EPI507495  EPI507496  EPI507497  EPI507498  EPI507499  EPI507500  EPI507501  EPI507502 |
