# Supplementary material for: An efficient genome sequencing method for equine influenza [H3N8] virus reveals a new polymorphism in the PA-X protein
Source: Virol J. 2014 Sep 2;11:159. doi: 10.1186/1743-422X-11-159 (PMC4161859; doi:10.1186/1743-422X-11-159)
Supplement: Supplementary file 2 — Additional file 2: GenBank accession numbers for A/equine/Richmond/1/07 and A/equine/Lincolnshire/1/07, and GISAID EpiFlu database [ [19]] accession numbers for other strains. (DOCX 14 KB) [file 12985_2014_2483_MOESM2_ESM.docx]

Additional file 2.

| Segment | Richmond/1/07 | Lincolnshire/1/07 |
| --- | --- | --- |
| 1  2  3  4  5  6  7  8 | KF559332.1  KF559333.1  KF559334.1  FJ195395.3  KF559335.1  KF559336.1  KF559337.1  FJ195429.2 | KF559338.1  KF559339.1  KF559340.1  FJ195398.2  KF559341.1  KF559342.1  KF559343.1  FJ195427.2 |
