# Supplementary material for: An efficient genome sequencing method for equine influenza [H3N8] virus reveals a new polymorphism in the PA-X protein
Source: Virol J. 2014 Sep 2;11:159. doi: 10.1186/1743-422X-11-159 (PMC4161859; doi:10.1186/1743-422X-11-159)
Supplement: Supplementary file 3 — Additional file 3: Alignment of predicted amino acid sequences for PA-X from EIV isolated in the UK between 2005 and 2013. Amino acids of the C-terminal PA-X domain only, following the +1 frameshift, are shown. (DOCX 15 KB) [file 12985_2014_2483_MOESM3_ESM.docx]

Additional file 3.

....|....| ....|....| ....|....| ....|....| ....|....| ....|....| .

10 20 30 40 50 60

**Lanark/05**  VSPREAKRQL KKDLKSQGRC ASLPITVSHR TSPALKILES MWMDSNRTAA LRVSFLKCPK K

**Wales/05**  .......... .......... .......... .......... .......... .......... .

**Essex/1/05**  .......... .......... .......... .......... .......... .......... .

**Essex/2/05**  .......... .......... .......... .......... .......... .......... .

**Lanark/06**  .......... .......... .......... .......... .......... .......... .

**Southampton/06**  .......... .......... .......... .......... .......... .......... .

**Lincolnshire/06**  .......... ........Q. .G........ .......... .......... .......... .

**Horsham/07**  .......... .......... .......... .......... .......... .......... .

**Maidstone/1/07**  .......... .......... .......... .......... .......... .......... .

**Southampton/1/07**  .......... .......... .......... .......... .......... .......... .

**Southampton/2/07**  .......... .......... .......... .......... .......... .......... .

**Richmond/1/07**  .......... ........*. .......... .......... .......... .......... .

**Richmond/2/07**  .......... ........*. .......... .......... .......... .......... .

**Newmarket/07**  .......... .......... .......... .......... .......... .......... .

**Cheshire/1/07**  .......... ........*. .......... .......... .......... .......... .

**Cheshire/2/07**  .......... ........*. .......... .......... .......... .......... .

**Cheshire/3/07**  .......... .......... .......... .......... .......... .......... .

**Lincolnshire/1/07**  .......... ....R..... .......... .......... ........N. .......... .

**Aboyne/2/08**  .......... .......... .......... .......... .......... .......... .

**Lanarkshire/1/08**  .......... .......... .......... .......... .......... .......... .

**Lanarkshire/2/08**  .......... .......... .......... .......... .......... .......... .

**Lanarkshire/3/08**  .......... .......... .......... .......... .......... .......... .

**Perthshire/3/09**  .......... .......... .......... .......... .......... .......... .

**Yorkshire/09**  .......... .......... .......... .......... .......... .......... .

**Dorset/09**  .......... .......... .......... .......... .....S..D. .......... .

**Lanarkshire/09**  .......... .......... .......... .......... .....S..D. .......... .

**Shropshire/10**  .......... .......... .......... .......... .......... .......... .

**Devon/1/11**  .......... .......... .......... .......... .......... .......... .

**E. Renfrewshire/2/11**  .......... .......... .......... .......... .......... .......... .

**Northamptonshire/1/13** .......... .......... .......... .......... .......... .......... .
